# Perspective: Connecting the dots between domestic livestock ownership and child linear growth in low‐ and middle‐income countries

**DOI:** 10.1111/mcn.13618

**Published:** 2024-01-08

**Authors:** Callum Lowe, Haribondhu Sarma, Darren Gray, Matthew Kelly

**Affiliations:** ^1^ Department of Applied Epidemiology, National Centre for Epidemiology and Population Health, College of Health and Medicine Australian National University Acton Australian Capital Territory Australia; ^2^ Population Health Program QIMR Berghofer Medical Research Institute Brisbane Queensland Australia

**Keywords:** animal faeces, animal source foods, child stunting, domesticated animals, environmental enteric dysfunction, linear growth faltering, low‐ and middle‐income countries

## Abstract

Child stunting due to linear growth faltering remains a pervasive issue in low‐ and middle‐income countries. Two schools of thought have existed pertaining to the role of domestic livestock ownership (DLO) in child linear growth. On one hand, it is argued that DLO leads to greater income and financial security, resulting in better child‐raising conditions, including greater animal‐source food (ASF) consumption, having protective effects towards child stunting. On the other hand, researchers argue that DLO contributes to faecal contamination and transmission of zoonotic enteric infections from animals to children, thus having destructive effects on child growth. Reviews of this association have revealed ambiguous findings. In this perspective, we argue that measuring the association between exposures to domesticated animals and child stunting is difficult and the ambiguous associations revealed are a result of confounding and differences in the management of DLO. We also argue that the increasingly prominent area of research of environmental enteric dysfunction, a sub‐clinical condition of the small intestine thought to be due to frequent faecal pathogen exposure and associated with stunting, will be a useful tool to measure the potential destructive effects of DLO on child growth. We present our argument and identify challenges and considerations and directions for future research.

## Key messages

- Research shows mixed effects of domestic livestock ownership on child linear growth in low‐ and middle‐income countries.
- Positive effects may flow on to improving child nutrition where animals and poultry are used for consumption or contribute to higher income and subsequent better child nutrition and home environment.
- Negative effects towards child growth occur where livestock is owned in such a way that infants are chronically exposed to animal faeces, which may result in environmental enteric dysfunction and diarrhoea.
- Stunting interventions must reduce infants' exposure to animal faeces whilst being sensitive to the role of livestock for the household economy.

## Graphical abstract

Domestic livestock ownership in low‐ and middle‐income countries can have positive or negative consequences for child linear growth. Safely managed animals that provide additional income and animal protein for households will improve child nutrition, but poorly managed animal faeces contribute to poor growth through repeated enteric infections.

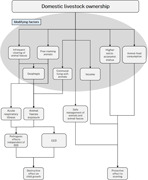

## 1 CHILD STUNTING, ENVIRONMENTAL ENTERIC DYSFUNCTION AND POOR WATER, SANITATION AND HYGIENE (WASH) CONDITIONS

Globally, over a quarter of children experience short stature, or stunting, defined by a height‐for‐age z‐score (HAZ) two standard deviations below the 2006 World Health Organization child growth standards median (Black et al., 2013). However, linear growth faltering during the first few years of life that leads to stunting to some degree is nearly universal in low‐ and middle‐income countries (LMICs) (Roth et al., 2017). Children that become stunted are more likely to have impaired cognitive ability, lower education, reduced earnings in adulthood and greater non‐communicable disease risk in adulthood (De Sanctis et al., 2021). These associations are not all causal but rather driven by exposure to the ‘deficient environment’ in infancy and early childhood (Leroy & Frongillo, 2019). As a result, child stunting prevalence provides an indicator of the degree to which the deficient environment is impairing children's growth and development in LMICs, and reduction in stunting will coincide with large economic gains (Akseer et al., 2022).

For decades, poor water, sanitation and hygiene (WASH) conditions have been recognised as a key factor behind child malnutrition, in particular, the large burden of diarrhoea attributable to poor WASH conditions in LMICs, five or more episodes of which are estimated to attribute to 25% of linear growth faltering (Checkley et al., 2008). Diarrhoea is also the second leading cause of death globally for children under 5 years old, highlighting its significance (World Health Organization, 2017). However, the failure of WASH interventions to prevent stunting and the observations that children experience catch‐up growth following bouts of diarrhoea has led researchers to question the notion that diarrhoea mediates the association between WASH and stunting (Humphrey, 2009). Whilst an ongoing area of research, it is being increasingly recognised that in poor WASH conditions, chronic infections of clinical or sub‐clinical nature lead to changes in the structure and function of the small intestine, most commonly defined by villous blunting and subsequently impaired absorption of nutrients, alongside crypt hyperplasia, microbial translocation and intestinal and systemic inflammation (Crane et al., 2015). These changes have been referred to as environmental enteric dysfunction (EED) to reflect the key role of the unclean environment. Research into EED has identified that it is a particularly strong predictor of linear growth faltering through prior research using tests of intestinal absorption and permeability (Lunn et al., 1991) and more modern methods of measuring the concentration of key biomarkers of EED in stool and serum samples (Harper et al., 2018). This remains a growing area of research with many unknowns.

## 2 DOMESTIC LIVESTOCK OWNERSHIP (DLO) IN LMICS

Domestic livestock and poultry living in close parameters with humans as a source of income and financial security is prominent in LMICs (Herrero et al., 2013). Livestock ownership has many positive benefits for communities, including greater empowerment of women. Ownership of animals leads to greater animal‐sourced food (ASF) consumption (Pasqualino et al., 2023) and increases dietary diversity, which reduces the risk of child stunting. However, animal faeces can pose a health threat to humans, in particular women, infants and young children who are at risk of a plethora of zoonotic infectious diseases such as *Campylobacter* spp., *Giardia intestinalis*, *Cryptosporidium* spp., Enterohaemorrhagic *Escherichia coli* and Shigatoxigenic *Escherichia coli* (Zambrano et al., 2014). Infection with such pathogens contributes to malnutrition, diarrhoea and intestinal damage and inflammation akin to EED that could lead to poor child growth (Guerrant et al., 2008). As a result, the effect of DLO on child nutritional status has been an active area of research.

## 3 ASSOCIATIONS BETWEEN DOMESTIC LIVESTOCK OWNERSHIP AND CHILD STUNTING

Two research directions have explored the relationship between DLO and child stunting. The first is that DLO can promote healthy child growth through increasing ASF intake and income (Mosites et al., 2015). On the other hand, DLO may worsen a child's nutritional status through increased transmission of zoonotic gastrointestinal pathogens from animal faeces to infants (Gelli et al., 2019; Penakalapati et al., 2017). Often studies are entirely focused on one of these directions, not considering the possibility of a positive and/or negative association in the same study site. Whilst there is a clear distinction between exposure to domesticated animals and exposure to domesticated animals' faeces, negative relationships between DLO and child growth have been found independent of exposure to animal faeces. For example, the work of George and colleagues in the Democratic Republic of the Congo found that caregiver reports of children merely touching domesticated animals was associated with lower HAZ score (George et al., 2021). As a result, the relationship between DLO and child stunting and growth as a whole is unclear. A recent systematic review by Zerfu et al. (2023) identified a significant volume of research identifying a positive association between livestock keeping and children's HAZ, but also many, albeit significantly fewer, papers finding neutral and inverse associations (Zerfu et al., 2023). Such ambiguous findings on the effect of exposure to animals/animal faeces were also briefly described in the review by Penakalapati et al. (2017). For example, an analysis from Mosites and colleagues found a 5% and 13% reduction in stunting prevalence in Ethiopia and Uganda, respectively, associated with a 10‐fold increase in household livestock ownership (Mosites et al., 2015). On the other hand, lower mean HAZ scores were associated with livestock ownership in multiple sites of sub‐Saharan Africa in a different study (Hetherington et al., 2017). Such ambiguous findings are problematic because it is not clear whether encouraging domestic livestock ownership in LMICs will have positive, neutral or negative effects on child growth. This is critical because population increase and greater disposable incomes among people in LMICs coupled with the nutrition transition will drive higher demand for domestic livestock production and ASF consumption. Thus, it is critical that a thorough understanding of its effects on child nutrition, particularly in these contexts where malnutrition rates are high, is paramount. Local livestock production in LMICs has the capacity to improve food security and alleviate significant malnutrition and poverty (Dominguez‐Salas et al., 2019). To reap the benefits of livestock ownership into the future whilst protecting maternal and child nutrition will require a consolidated understanding of these associations. In this perspective, we propose that the ambiguous findings are a result of differences in study designs/contexts and missing quantification of key confounding factors such as ASF consumption, socio‐economic status (SES), direct exposure to animal faeces and EED. We argue that EED should be used as a key indicator in future studies to assess the risk of livestock ownership to child nutrition.

## 4 EXPLAINING AMBIGUOUS ASSOCIATIONS BETWEEN DOMESTIC LIVESTOCK OWNERSHIP AND CHILD STUNTING

**Figure 1:**
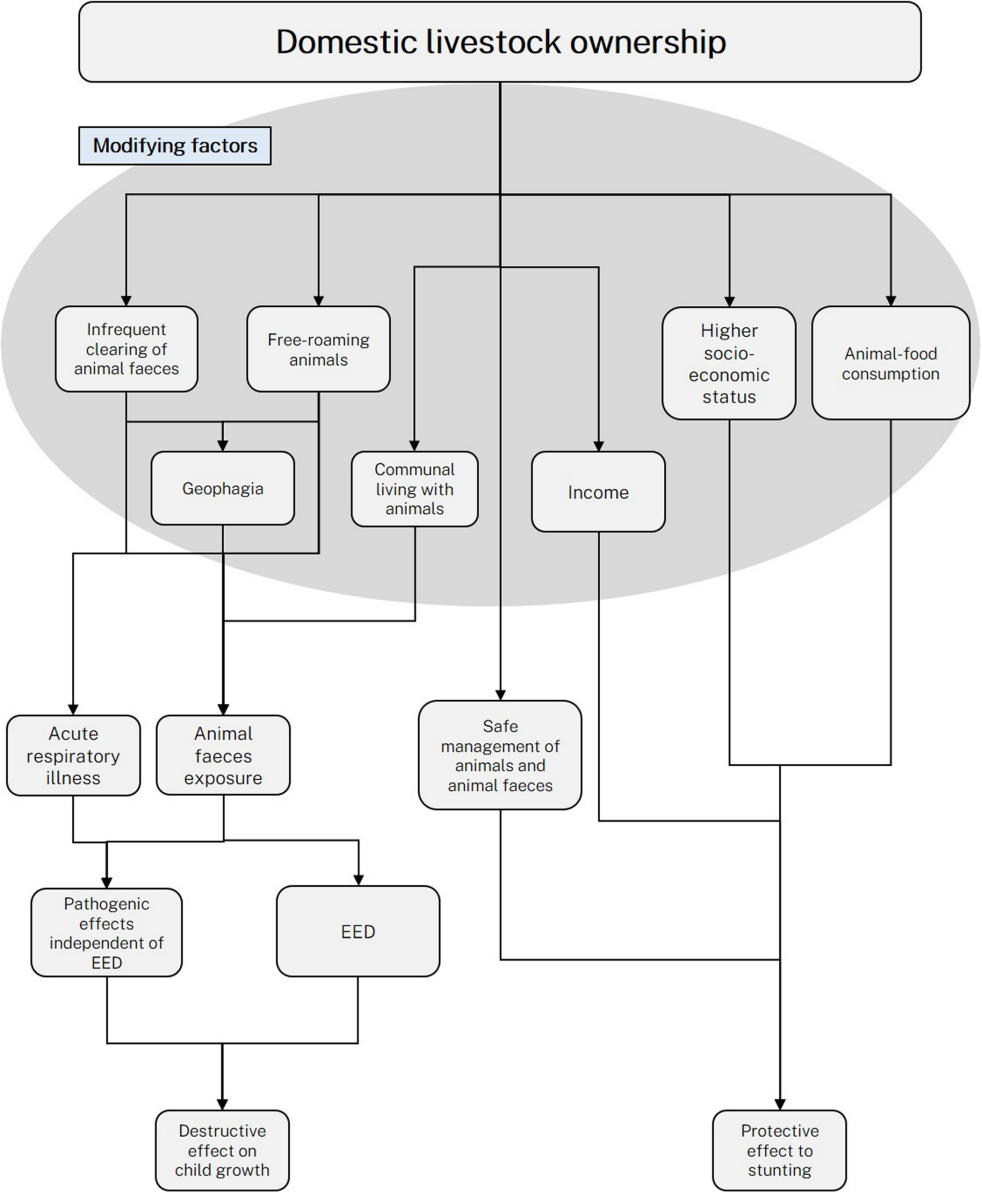
Proposed theoretical framework linking livestock ownership and child stunting through confounding and mediating factors. EED, environmental enteric dysfunction.

How can ownership of livestock be associated with positive gains in child HAZ in some studies, whilst in other studies associated with deficits in HAZ? The interplay of these factors is presented in Figure 1, which highlights the difficulty in measuring this association. The pathway linking ownership of livestock to child stunting may be confounded by SES. Households that have livestock and livestock in greater quantity would have more disposable income to spend. This would correlate with higher SES that predicts better child growth, likely through greater maternal education, maternal height/nutrition, better raising conditions and greater dietary diversity. Furthermore, ownership of livestock has been shown to lead to greater animal source food consumption among infants (Pasqualino et al., 2023), which has been demonstrated in some studies to have protective effects against stunting (Headey et al., 2018), although the exact mechanisms of this are not clear. Whether studies report a positive or negative association between livestock ownership and child growth may depend on the types of livestock owned. In the review by Zerfu and colleagues, it was found that cattle ownership was more often found to have a positive association with human nutrition compared to poultry ownership as a proportion of included studies measuring each association (Zerfu et al., 2023). Households that raise cattle are more likely to consume dairy milk, which has a particularly strong association with child growth; children raised in dairy milk‐producing households have a 0.52 higher HAZ score compared to children who are not, although this does reduce exclusive breastfeeding (Choudhury & Headey, 2018). Cattle may not come into direct contact with infants or with the domestic household environment, thus presenting only a small risk for infants encountering their faeces. It is unlikely that cattle ownership improves child growth through meat consumption, as meat is particularly expensive in LMICs and consumed irregularly, which would therefore not provide a consistent opportunity for improvements in child growth. Poultry ownership may increase egg consumption in only a small proportion of households. Whilst eggs are beneficial for child nutrition and supplementation trials demonstrate positive effects on child growth (Mahfuz et al., 2020), they may not be consumed in households because they are often sold in preference of direct consumption within the household (Morris et al., 2018) which is also driven by the fact that compared to dairy milk for example, eggs are less perishable. As to be discussed, poultry tend to roam freely and present a greater risk of transmission of enteric pathogens compared to cattle or other livestock.

Clearly, not in all cases does owning livestock show a positive association with child growth. Under other circumstances, such as where ownership of livestock is not in such a great quantity to be associated with SES or used for local ASF consumption, and where domesticated livestock contribute to faecal contamination of infants' environment with infrequent clearing of faeces, infants might be at greater risk of chronic exposure to faecal pathogens. In studies that report a positive association between livestock ownership and child growth, animals may have been kept away from child play spaces, thus reducing exposure to animal faeces and preventing EED and other zoonotic gastrointestinal infections. On the other hand, studies reporting a negative association may be conducted in conditions where animal faeces were poorly managed and animals were free to roam and defecate in the household and infants' play space.

We base these arguments on increased evidence that, in many cases, livestock ownership leads to greater faecal pathogen exposure among infants and subsequent growth faltering when livestock are unsafely managed.

## 5 CIRCUMSTANCES WHERE LIVESTOCK OWNERSHIP IS ASSOCIATED WITH STUNTING

Evidence suggests that certain practices in domestic livestock management predispose infants to a greater risk of stunting. Children with animals in their sleeping space had a 0.37 deficit in HAZ (95% confidence interval [CI] = −0.71, −0.04, *p* < 0.05) compared to those that did not in a study in urban Bangladesh (Monira et al., 2020). The REDUCE study of children under 5 years of age in the Democratic Republic of the Congo observed a 0.33 HAZ reduction in children who touched guinea pigs (95% CI = −0.58, −0.08, *p* < 0.05) and a 0.34 HAZ reduction in children who touched rabbits (95% CI = −0.64, −0.04, *p* < 0.05) (George et al., 2021). The study also found a 0.41 HAZ reduction among children with faeces (of animal or human origin) in their sleeping space (95% CI = −0.74, −0.09, *p* < 0.05). An observational study found a significant association between observation of animal faeces in the household environment and HAZ score among infants in Bangladesh (HAZ difference = −0.13, standard error [SE] = 0.07, *p* < 0.05) and Ethiopia (HAZ difference = −0.19, SE = 0.08, *p* < 0.05) but not Vietnam. A particularly interesting case of this ambiguity was demonstrated in the work of Headey and Hirvonen (2016). This research in rural Ethiopia found that whilst poultry ownership in households had a positive effect on child growth (0.29 difference in HAZ), corralling poultry overnight was associated with a 0.25 reduction in HAZ (*p* < 0.05) (Headey & Hirvonen, 2016). Another key case of conflicting results was observed in Eastern Chad, where the increasing number of cattle in villages was associated with greater rates of child wasting, but this effect was not seen in villages that reported regularly cleaning water transport containers (Marshak et al., 2016). In these instances, domestic livestock may have been managed poorly such that infants came into greater contact with animal faeces in their sleeping room and in their household environment.

## 6 ASSOCIATIONS BETWEEN DOMESTIC LIVESTOCK OWNERSHIP AND ENVIRONMENTAL ENTERIC DYSFUNCTION

We argue that in research studying the potential risk of domestic livestock towards child nutrition, the use of EED as an outcome provides a more direct measure as it is more proximal to the risk factor than stunting status. This will require less exhaustive effort to control for SES, ASF consumption and other potential confounding factors. There have already been some insights made in this area. Having an animal corral in the sleeping room of Bangladeshi infants’ home was associated with greater EED severity as measured by faecal neopterin (an indicator of immune‐cell activity in the small intestine), myeloperoxidase (an indicator of intestinal inflammation) and alpha‐1‐antitrypsin (an indicator of protein‐losing enteropathy) biomarkers (*p* < 0.05 for all), as well as higher odds of stunting (odds ratio [OR] = 2.53, *p* < 0.05) (George, Oldja, Biswas, Perin, Lee, Ahmed, et al., 2015). An association between keeping chickens indoors at night and EED was found in rural Ethiopia, although the association was not statistically significant (adjusted odds ratio = 1.95, 95% CI = 0.86–4.57, *p* = 0.11) (Chen et al., 2021). Among rural Malawian children with EED characterised by a higher lactulose‐to‐mannitol ratio (an indication of poor intestinal absorption and high permeability), 42% were in households with animals that slept indoors compared to 29% among infants with no EED (*p* < 0.05) (Ordiz et al., 2016). These studies provide strong evidence that domestic livestock kept indoors overnight, as an indicator of greater animal faecal pathogen exposure, contribute to stunting through EED. Greater research is needed however in other potential routes, for example, through infants playing in areas that are trespassed by animals. Some indication of this has been found in studies of geophagia, the act of mouthing soil, which has been reported to be associated with EED in rural Bangladesh (George, Oldja, Biswas, Perin, Lee, Kosek, et al., 2015). Infants have been observed to frequently mouth chicken faeces as part of exploratory behaviour in contaminated environments (Ngure et al., 2013), which may also contribute to EED and subsequent growth faltering. An area of research that has had little attention is whether pregnant women with frequent contact with animals and their faeces can develop EED as a result of frequent enteric infections, which have been demonstrated to increase the risk of low‐birthweight babies (Chan & Smith, 2018), a key predictor of stunted growth.

## 7 ENVIRONMENTAL SURVEYS OF MICROBIAL CONTAMINATION FROM ANIMAL FAECES

The role of exposure to animal faeces in contributing to EED is supported by evidence of the microbial contamination on infants, their mothers and in the household environment of households that own livestock. In an exploratory study in rural Bangladesh by Ercumen et al. (2020), high *E. coli* contamination of water, soil, food and hands was found, and the presence of animals in the home compound was associated with higher counts of *E. coli* on soil, stored water and food (all associations *p* < 0.05), in particular, chickens (Ercumen et al., 2020). A systematic review and meta‐analysis estimated that domestic exposure to poultry is associated with *Campylobacter* infection (OR = 2.73, 95% CI = 1.90–3.93) and any animal associated with EHEC/STEC infection (OR = 4.80, 95% CI = 2.42–9.48) (Zambrano et al., 2014). Furthermore, 20 out of 29 included studies identified an association between domestic animal exposure and diarrhoea, a well‐established cause of child stunting (Checkley et al., 2008). Budge and colleagues demonstrated that domestic animal husbandry contributed to bacterial contamination of infants' hands and floor surfaces (Budge et al., 2019).

Given these examples, we urge the importance of research studies investigating the effects of domestic livestock ownership to consider elements of livestock management, including whether animal faeces were kept away from infants' play space and sleeping space. Furthermore, directly measuring if infants come into contact with animal faeces (e.g., through geophagia or direct contact) through caregiver observations is critical for establishing risk. The need for detailed estimates of exposure to animals and their faeces was highlighted in a recent review indicating that the majority of studies evaluating human exposure to animal faeces used poor‐quality measurements (Ballard et al., 2023).

## 8 CONSIDERATIONS REGARDING THE USE OF EED AS AN INDICATOR

It should also be noted that it is possible that exposure to animal faeces may contribute to child stunting independent of EED. It is thought that some zoonotic pathogens, such as *Giardia*, can contribute to child stunting independent of elevated intestinal or systemic inflammation, but possibly through causing amino acid deficiencies (Giallourou et al., 2023). This highlights the importance of measuring other indicators of exposure and pathology from zoonotic faecal pathogens such as micronutrient deficiencies and direct identification of pathogens in stool for example. As illustrated in Figure 1, exposure to poultry and birds among infants may also contribute to growth faltering through acute respiratory illness. Parvin and colleagues demonstrated that young children in urban Bangladesh were more likely to have respiratory symptoms if they lived with chickens or birds (Parvin et al., 2022). Respiratory illness may then contribute to growth faltering through appetite suppression and chronic systemic inflammation (Syed et al., 2018). This remains a relatively unexplored area and warrants future research.

## 9 CONSTRAINTS AND CONSIDERATIONS TO THE SAFE MANAGEMENT OF DOMESTICATED ANIMALS IN LMICS

The safe management of animal faeces is not as simple as it sounds. In LMICs, women and men may not have the time to engage in frequent clearing of animal faeces from the environment as they juggle other responsibilities. The purchase of animal cages or fenced areas for animals to breed may not be possible in conditions of poverty. Furthermore, close interaction between infants and animals may serve as a source of exploration, activity and learning in the absence of more expensive toys and games. The qualitative work of Ngure et al. (2019) highlighted some of the economic constraints in the safe management of domestic livestock, finding participants reported that persons with the economic means could afford to build a fenced chicken house, whereas poorer persons might have to allow chickens to roam free (Ngure et al., 2019). Free‐roaming animals such as chickens can scavenge for their own feed and thus are more favourable for owners with limited income. Further understanding of this is of critical importance as mothers of infants do not always recognise that the faeces of animals present a health risk to humans (Ellis et al., 2020; Marquis et al., 1990). There may also exist a belief that free‐roaming animals grow better (Marquis et al., 1990). Animals may be corralled indoors at night to prevent theft. After infants learn to crawl, exploration of the environment is a core part of infant development as they learn about objects, animals and their interaction with the environment. Adequate stimulation in this regard is critical for child development, but in settings where DLO and animal faeces are managed poorly, these exploratory behaviours may be one of the largest contributors to infection with enteric pathogens. Interestingly, some research has shown that positive associations between DLO and child growth only occur when domestic livestock is managed by women, and not men (Jin & Iannotti, 2014). This has been observed even independent of the contribution of male‐headed households having higher income (Johnson & Rogers, 1993). It may be that women are better inclined than men in ensuring the benefits of DLO transfer to child health and nutrition (Jin & Iannotti, 2014).

## 10 FUTURE DIRECTIONS

Moving forward, we encourage greater research to investigate EED in measuring the association between exposure to animals and their faeces with child stunting and to consider confounding factors including ASF consumption and differences in household income and SES. Whilst an evolving field, great strides have been made in measuring EED from invasive intestinal biopsies to troublesome dual sugar absorption tests and now to the mainstay of quantification of noninvasive tests of stool and serum biomarkers of the domains of EED. A key area of future research is to better understand what specific aspects of animal/livestock ownership (such as cohabitation with infants, corralling animals indoors overnight, and infrequent clearing of their faeces) present the greatest risk to child nutrition through EED. We make the following recommendations for future research:

- Studies investigating child nutrition outcomes through consumption of animal‐source foods should consider quantifying exposure to animals and their faeces as well as biomarkers of EED where appropriate.
- Studies investigating the link between exposure to animals and their faeces with child stunting consider measuring animal source food consumption and SES as well as biomarkers of EED.
- Future research investigates the conditions in which having domestic livestock exacerbates EED among infants versus conditions that do not lead to EED or chronic faecal pathogen exposure among infants.
- Explore barriers to and caregiver attitudes towards the safe management of domesticated livestock and their faeces, with particular attention to pathways of exposure among infants.
- Develop interventions for the management of animal faeces and infants' play environment that does not reduce critical exploratory behaviours or have destructive effects on parents' ability to own livestock.

Research investigating these issues will permit a better understanding of the circumstances in which domesticated livestock ownership will contribute to better child health outcomes and on the other hand contribute to faecal pathogen exposure among infants that could lead to child stunting through EED. Findings from studies of this kind would be crucial in developing effective interventions that are both culturally sensitive and recognise the significance and contribution of livestock ownership to local economies in LMICs.

## AUTHOR CONTRIBUTIONS

Callum Lowe, Matthew Kelly, Haribondhu Sarma and Darren Gray conceptualised the manuscript. Callum Lowe wrote the first draft of the manuscript. Callum Lowe, Matthew Kelly, Haribondhu Sarma and Darren Gray reviewed and edited the manuscript draft.

## CONFLICT OF INTEREST STATEMENT

The authors declare no conflicts of interest.

## Data Availability

Data sharing is not applicable to this article as no data sets were generated or analysed during the current study.
